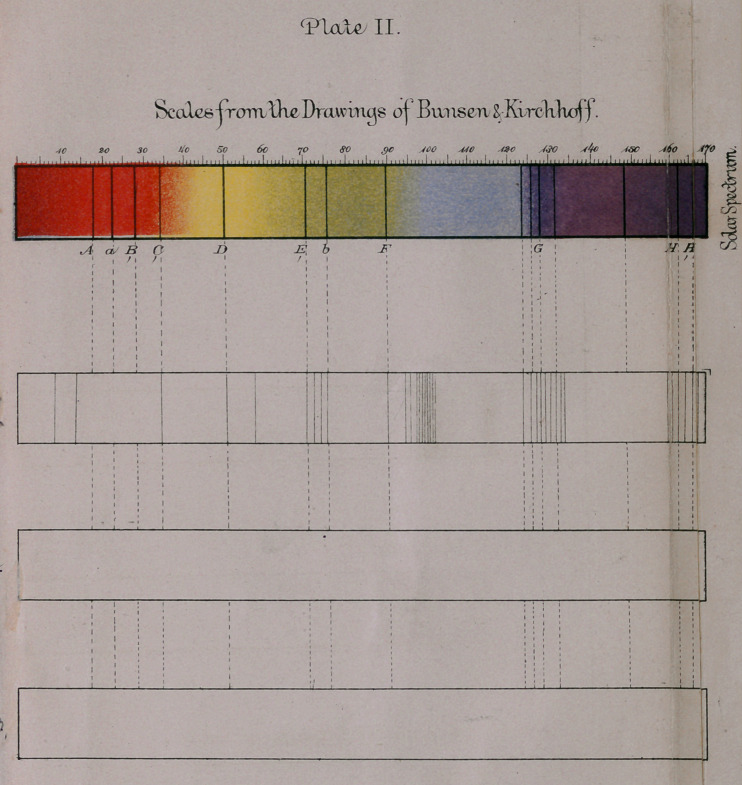# Spectrum Analysis of Blood*Read before the Buffalo Medical Club.

**Published:** 1879-08

**Authors:** W. H. Pitt


THE BUFFALO

MEDICAL AND SURGICAL JOURNAL.

VOL. XIX.—AUGUST, 1879.—No. 1.

### Original Communications.

### SPECTRUM ANALYSIS OF BLOOD.*

BY W. H. PITT, M. D.

THE advance made within a few years in molecular physics through a better knowledge of radiant light, affords a guide in investigations which lead to a clearer understanding of the phenomena of life.

Vague ideas and traditional notions have been abandoned for the more rational view which science points out in dealing with the growth and decay of organisms. The physicist and physiologist knowing this have only to do with facts, and so far as actual knowledge is concerned their interpretation. The days of speculative philosophy and the enchantments of classical story, have passed away. It is no wonder that, listening to illusions all up the restless ages, it took man so many generations to find out any thing positive of himself or his surroundings.

The discovery of the circulation of the blood by Dr. Harvey marked a new era in human progress. It is sad to think that up to his time so little had been done for physiology. But with its extensive literature, and illustrations drawn from every source of

* Read before the Buffalo Medical Club.

the animal and vegetable kingdom there is much unknown, and which demands careful study. Chemical analysis and microscopical observation, however, have accomplished about all that we can expect in determining the composition of the blood and its different aspects. To the spectroscope which is comparatively new and not in general use, we must look, year by year, for further information.

In the examination of blood, which is here submitted for your consideration, I have employed an instrument of four dense flint glass prisms. They have the advantage of widely separating the D line, and in giving broader absorption bands than those which are commonly seen in chromolith. plate. My first experiment consisted in putting a drop or two of arterial blood in a glass cell containing distilled water, in order to secure the absorption bands which are shown in plate I, strip 1. These are the well-known signs of aerated blood when diluted with water, or, as we shall see hereafter, when examined during circulation in the arteries. Strip 2, plate I, is a solution of blood and magenta, in which we see the blood bands are completely occluded. After adding sulphite of soda the wide overshadowing band due to the aniline red disappears, and the others remain unchanged. See plate I, strip 3. It is well known that a solution of cochineal and alum affords bands very similar to blood, as they occupy nearly the same spectral space, and are likely to deceive the unpracticed eye. A little boric acid added increases their refrangibility, and if blood be present it can be detected as before indicated.

Some vegetable reds also display absorption bands similar to blood; but they are always removed on addition of ammonia or a chemical re-agent which will not act upon blood—sulphite of potash, for instance. Generally speaking it is safe to conclude, and I am not aware that there is a single exception to this rule, that if the proper precaution be taken, the absorption bands of the red corpuscles may always be restored. So that, according to these and similar tests, no matter what the coloring matter in solution, the spectroscope can absolutely determine either the presence or absence of blood.

I must not dwell, however, upon a part of the subject which has been so ably investigated in all its details by more competent observers.

The object of this paper is to bring forward a few results in spectrum analysis, which, as far as known, are here for the first time described.

The question referred to the spectroscope to answer was: "What are the elementary constituents of blood vapor in a Torricellian vacuum?" Toward a satisfactory solution a Geissler tube, open at one end, was connected with a glass tube thirty-four inches long, and the whole arrangement filled with mercury. After inverting and opening the end under mercury the column settled down till checked by atmospheric pressure. A few drops of fresh arterial blood were then introduced, and rose to the surface of the mercurial column; and of course was free to vaporize and fill the vacuum. The tube containing the vapor was then hermetically sealed and detached by the blowpipe. On sending a current of electricity through, of sufficient tension to make it highly luminous, I brought it before the slit and with a comparison spectrum, located according to coincidence the following lines, which see plate II, strip 1. It will be noticed that the oxygen, hydrogen, and nitrogen lines are brought out, and also that there are some bands which are possibly due to nitrous oxide. The spectrum is somewhat complex, and the greater portion of it slightly luminous. Now, whether the oxygen comes from solution, from carbonic acid, or from aqueous vapor, I am unable to say.

But the two hydrogen lines undoubtedly come from water, while the nitrogen might be naturally suspected as escaping from solution. At some time not far distant, I hope to arrive at something more positive in reference to these gaseous lines. By testing the oxyhaemoglobin alone in a Torricellian vacuum, I may be able to announce the comparative chemical affinity or adhesion which exists between the red corpuscles and oxygen. Should venous blood be subjected to a similar process in a vacuum, I am inclined to the opinion that the carbonic acid which it holds could be more accurately determined than by the usual methods of analysis. To answer fully, however, many points which are naturally suggested in reference to these gases in the blood will take time and patience, as they involve experiments of a most delicate nature. Since there are always sources of error in quantitatively determining soluble gases when experimenting in the open air, the method indicated above would lead, I think, to closer figures than those which we have been accustomed to accept.

It should be remarked that in all examinations which refer to the two absorption bands located between the sodium line D on the borders of the orange and the yellow, and the hydrogen line E in the green, we cannot tell whether the blood be from man or some other vertebrate animal. Certain it is, however, that two specimens may give shades of differences; but these effects probably arise from the continued exposure of the retina to the spectrum colors. After watching for hours through an intense light phantom bands will sometimes appear and fade away. This occurs from the fact, no doubt, that the genuine bands are for the most part dark, and are complimentary to white light; or, that the eye is sympathetic to impressions made upon it for a long time even when closed. There is still another method of observing which cannot be relied upon, and which I beg to call attention to in the following example:

After one of Dr. Mason's lectures, the blood from a dog and a rabbit were taken. One of these animals was poisoned by woorara and the other by strychnia. With a desire, and I may add longing to detect if possible a difference in their bands either in width, intensity, or some other variation, I attached the cells to a slide and brought one and then the other into the field of view. They did not appear alike. But this was only an illusion, as next day when the eyes were rested and a double spectrum used their bands coincided in every particular. I am not aware that notice has been directed to these points by others, and give them merely as my own experience and for what they are worth. But while these bands are persistent in very weak solutions, even as 1 to 7,000 they may be plainly seen in the mesentery of the living animal, or in the web of the frog's foot. Acting upon this latter hint, I endeavored to get the same from some part of the circulation and thus study the oxyhaemoglobin portion of the blood in the arteries of the human body. For this purpose I used a large bi-convex lens, and brought the direct sunlight to a focus on the instrument—or rather a little out of focus so as not to burn the hand.

The thumb and first finger were then extended apart as far as possible, and the web of the obtuse angle, which held to the light is seen to be translucent, brought into the cone of light close to the slit. By taking these precautions, I have been able to study the absorption bands of the arterial blood in the hand for hours. If the hand, as above directed, be held firmly to the slit, and the radial artery be pressed by the other hand, or by an assistant, they fade a little but on releasing the pressure they again resume their natural width and shade. Carbonic acid or hydrogen inhaled has also about the same effect. When the wrist is tightly ligated for a few minutes they almost entirely disappear; but on cutting the ligature are quickly restored. In a small recitation room in whiich a hundred pupils had been assembled they were changed in a marked degree, but as soon as the assistant let in upon me the air through the open windows, they brightened up in a few inspirations.

The skin at the sub-acute angle of two adjacent fingers may also be used with the same results; but the wide and wedge-like space between the thumb and first finger is to be preferred mostly from convenience. This furnishes an easy way to study the red corpuscular condition of individuals of different temperaments, and in different states of health. It only remains to map the spectra as a guide to go by, and deduce from actual experiment the absorption bands of blood diseases in fevers, &c. And this can be done by the method I have indicated without drawing a drop of blood from the patient. As I have said before this idea of using transmitted light from the human body in examinations with the spectroscope, was suggested by the common experiment which all are so familiar with in studying the circulation in the frog's foot. I am aware, however, that Herr Verordt, a German physiologist, published last summer the fact that these bands may be seen by *reflected* light—that is to say by holding two fingers together and admitting the light between their two surfaces. I have repeated his experiments with reflected light not only from the fingers but from other surfaces of the body.

They do not give, however, the satisfactory results which *transmitted* light from the blood vessels affords, and which, so far as I know, is here for the first time described to the profession.

## Figures and Tables

**Plate I. f1:**
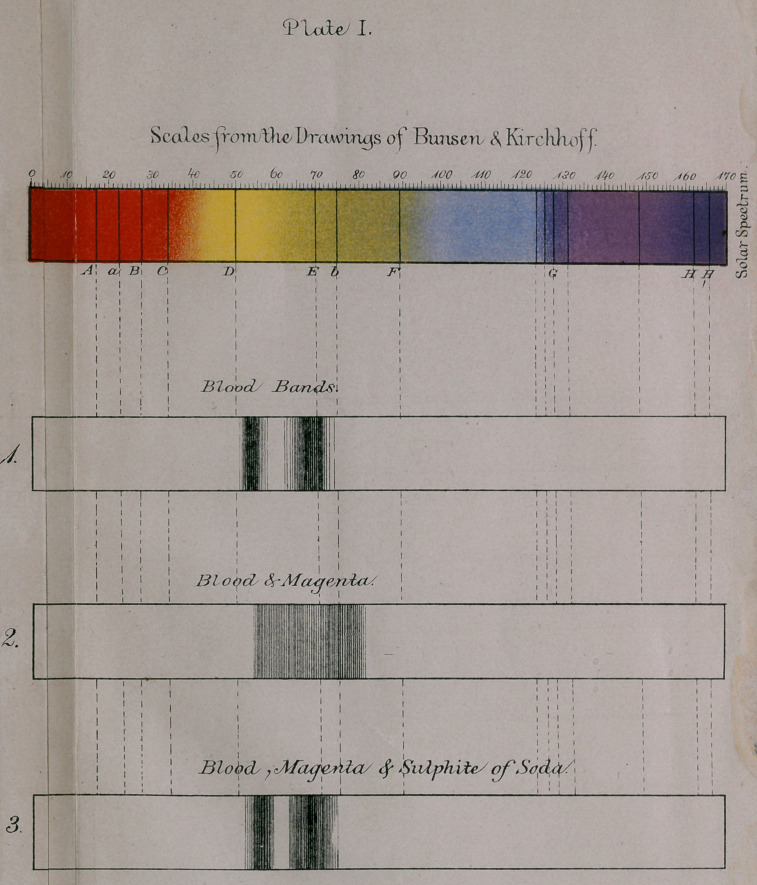


**Plate II. f2:**